# An Unusual Presentation of Glandular Fever

**DOI:** 10.1155/2022/5981070

**Published:** 2022-03-18

**Authors:** Dominic Worku, Li Hui Chang, Ian Blyth

**Affiliations:** ^1^Infectious Diseases, Morriston Hospital, Swansea, UK; ^2^Public Health Wales, Cardiff, UK; ^3^Gastroenterology, Morriston Hospital, Swansea, UK

## Abstract

Epstein-Barr virus (EBV) is an ubiquitous DNA herpesvirus with >90% of adults >40 years of age showing a serological response. While in their youth, primary EBV infection may pass unnoticed, young adults have a high incidence of infectious mononucleosis (IM). This is characterized by a triad of pharyngitis, cervical lymphadenopathy, and fever because of a self-limiting lymphoproliferative disease. Common complications include but are not limited to hepatitis, splenomegaly, encephalitis, and haemophagocytic lymphohistiocytosis (HLH) with evidence that Caucasian males and smokers are more likely to suffer severe disease. Here we present a 21-year-old male who presented with a 2-week history of fever, dry cough, and a 4-week history of pharyngitis. He had no exposure to unwell contacts and denied any new sexual partners. Examination revealed general pallor with tender bilateral cervical lymphadenopathy and pharyngeal erythema. Admission bloods revealed pancytopenia (WCC 1.5 × 10^9^/L, Plt 84 × 10^9^/L, and Hb 82 g/L) with normal reticulocyte count and raised mean corpuscular volume (114 fL). Serum vitamin B12 and folate were low with serum ferritin raised (1027 *µ*g/L) suggesting a proinflammatory state. Admission liver function tests, coeliac serology, autoimmune panel (ANA, ANCA, and anti-dsDNA), hepatitic (hepatitis A, B, and E), human immunodeficiency virus (HIV), toxoplasmosis, parvovirus, and CMV serology were normal. The monospot test on day 1 of the presentation was negative. Ultrasound (US) of the abdomen on day 3 of the presentation revealed isolated splenomegaly (16.8 cm). Day 4 EBV serology (VCA IgM, VCA IgG, and EBNA IgG) was negative as such haematological investigations including JAK2, serum free light chains, and BCR-ABL were undertaken alongside cervical lymph node core biopsy. Repeat Monospot testing on day 7 came back positive. Repeat EBV serology now showed equivocal EBV VCA IgG (0.77 OD) and positive VCA IgM (9.04 OD) with concurrent new hepatitis. Histopathology of the core biopsy revealed Sternberg-reed cells and a mixed immunoblastic reaction in keeping with resolving IM. This case highlights the need for physicians to have a strong clinical suspicion of IM and understand the multiple ways in which IM may be present as well as the time lag to positivity in serological testing.

## 1. Introduction

Epstein-Barr virus (EBV) is a linear double-stranded DNA gamma herpesvirus also known as human herpes virus 4 [1–4]. It has a capsid surrounded by a tegument and an envelope embedded with glycoproteins [5]. Throughout the world, ∼90% of adults by age 40 years show serological response to EBV although this is dictated by geography, ethnicity, and race [1, 6, 7]. The primary method of spread is through contact with respiratory secretions; however, cases of dissemination via sexual intercourse, blood transfusion, and allograft transplantation have been recorded. The incubation period of EBV infection is between 4 and 7 weeks, with a peak infection rate in the midteenage years primarily due to changes in social behaviour including kissing [2, 6, 8].

In the first steps of infection, EBV affects the epithelial cells of the oropharynx. This occurs through direct fusion of the viral envelope with the cell plasma membrane. After replication within the epithelial tissues, the virus is primed for entry into primarily B-cells but also multiple other cell types. For B-cell infection, this requires the use of viral gp350/220 to CD21 while gp42 binds to major histocompatibility complex 2 to initiate entry. Upon binding of the virion with the endosomal membrane viral tegument proteins are released into the host B-cell including BNRF1 and BZLF1 leading to activation of transcription and lytic gene expression this accompanied by apoptosis suppression via BHRF1 and BALF1 leading to a hyperproliferative state occurs akin to a germinal centre reaction [4]. Viral shedding within the oropharynx can be very high and may persist for many months/years postprimary infection with median EBV viral loads of 63,000 copies/mL of saliva recorded [6]. Following the proliferative phase, germinal centre B-cells leave this cycle to form latently infected memory B-cells which cannot be eradicated by the immune system, leading to the future risk of reactivation. Due to this persistence, it is not surprising that EBV is capable of both *in vitro* and *in vivo* transformation of mammalian cells and was the first oncogenic virus identified.

Globally, EBV is implicated in ∼200,000 malignancies each year including Hodgkin's disease, nasopharyngeal carcinoma, and Burkitt's lymphoma [1]. In pathologies such as Burkitt's lymphoma, latently infected B-cells fail to successfully develop into memory B-cells leading to continued immune hyperstimulation. Increasingly, the role of EBV in other disorders is being appreciated including neuroinflammatory disorders (multiple sclerosis), autoimmune diseases (such as rheumatoid arthritis and systemic lupus erythematosus) and possibly chronic fatigue syndrome; however, the mechanisms of this are still yet unexplained [8, 9]. There is a growing awareness of chronic active EBV infection, particularly in Asia, where it is commonest and the treatment for this includes stem cell transplantation [10]. Given the inherent risks of future lymphoma, particularly in immunosuppressed individuals, a key objective has been the development of a vaccine that might be able to attenuate the T-cell responses that cause IM and prevent related disease [5]. Trials of tetrameric gp350 constructs induce high levels of neutralising antibodies in murine models with initial human trials showing promising results [5].

While primary EBV infection in children is often asymptomatic, this is not the case in adolescents, of whom up to 70% present with infection mononucleosis (IM), colloquially known as glandular fever. Overall, in young adults, IM occurs 1/1000 per year versus 1/50,000 in the general population [11]. IM is characterized by the triad of pharyngitis, cervical lymphadenopathy, and fever which can be found in other common conditions (Table 1), during which time a self-limiting lymphoproliferative disease state is present with up to 20% of all B-cells becoming infected with exaggerated CD8 T-cell responses leading to symptom development [12]. In comparison, in healthy carriers, 1 in 50 million leucocytes are EBV infected [13].

Severe important sequelae can occur however in both immunocompetent and immunocompromised hosts including meningoencephalitis, splenic rupture, aplastic anaemia, acalculous cholecystitis, and hemophagocytic lymphohistiocytosis (HLH) and thus EBV infection can present atypically and present a diagnostic challenge physicians [5, 14]. While IM in ∼90% of cases is due to EBV, 5–16% of cases are due to cytomegalovirus a dsDNA betaherpesvirus which in immunocompromised individuals can cause multisystem diseases and congenital malformations [5, 15]. Although there is disagreement among studies, there appears to be slightly altered epidemiological and clinical features. In those with IM caused by CMV, patients appear to be 10–15 years older, have milder lymphadenopathy, but more serious hepatitis and thrombocytopenia [16, 17]. One key discriminatory symptom between EBV/CMV associated IM includes facial oedema, particularly around the eyes, which is specific to EBV due to disturbances in orbital lymphatic drainage (Hoagland Sign) and is often underappreciated [18].

In this case report, we present a 21-year-old homosexual man who presented with pancytopenia and a 4-week history of night sweats with cervical lymphadenopathy and negative viral screens which led to a diagnostic dilemma. Through repeated testing for EBV, seroconversion was demonstrated and a diagnosis was made.

## 2. Clinical Presentation

A 21-year-old male with a history of migraines presented to Morriston Hospital in August 2021 following a month's history of pharyngitis and odynophagia and a 2-week history of fever, night sweats, and dry cough which had not responded to amoxicillin therapy. He denied any significant weight loss and no recent sexual partners. There was no history of exposure to unwell contacts or any relevant family history including that for autoimmune disease. He had not left the UK in the preceding 5 years, and he owned no pets, but was in regular contact with his sister's cat.

At presentation, clinical examination revealed ongoing pyrexia (>39°C), general pallor with tender bilateral submandibular lymphadenopathy. There was no evidence of inguinal lymphadenopathy, genital ulceration, or rash. Examination of the pharynx revealed no exudate, mild trismus alongside ulceration of the soft palate and tongue with hyperaemia. Admission blood tests revealed pancytopenia (Hb 82 g/l, WCC 1.5 × 10^9^/L, Plt 84 × 10^9^/L, and neutrophils 1.0 × 10^9^/L), macrocytosis (MCV 114 fL), normal reticulocyte count, and raised CRP (30 mg/l) with normal kidney and liver function tests. Coagulation tests revealed prolonged prothrombin time of 13.4 seconds with raised fibrinogen (4.5 g/L). Blood film confirmed pancytopenia with no blast cells noted, hypersegmented neutrophils, and occasional teardrop poikilocytes. Serum vitamin B12 (167 ng/L) and folate (<2.0 *µ*g/L) were very low and replacement was initiated. Serum ferritin was raised to 1027 *µ*g/L (15–300 *µ*g/L) highlighting a proinflammatory state. Coeliac IgA TTG, ANA, ANCA, and anti-dsDNA titers were normal. Further history revealed no abnormal bleeding and an extremely poor diet prompting a further micronutrient screen to be sent.

Initial glandular fever screening, utilizing a *monospot* test (day 1 of presentation), was negative. Subsequent microbiological investigations to rule out important mimics including acute retroviral syndrome were performed. Hepatitis A, B, and E, syphilis, Q-fever, toxoplasmosis, Whipple's, parvovirus, cytomegalovirus (CMV), and human immunodeficiency virus (HIV) antibody tests were negative. Respiratory viral swabs including adenovirus and SARS-CoV2 respiratory PCR screens were negative, alongside negative peripheral blood cultures. Ultrasound (US) of the abdomen (day 3) revealed splenomegaly of 16.8 cm but no hepatomegaly or intraabdominal lymphadenopathy.

Due to ongoing neutropenia with pyrexia and rising inflammatory markers (CRP 83 mg/L) and to protect from bacterial gut translocation, coamoxiclav and ciprofloxacin therapy were commenced. Computed tomography of the thorax, abdomen, and pelvis was subsequently performed (day 7), which highlighted marked splenomegaly only with no other intraabdominal findings and suggested possible haematological disease. JAK2, serum-free light chains, and BCR-ABL screens were normal; however, LDH was raised to 515U/l (<250u/L).

EBV serology (VCA IgM, VCA IgG, and EBNA IgG) was negative on day 4 of admission; however, on day 7, repeat *monospot* testing became positive, and EBV serology was resent. At this time, due to a lack of a unifying diagnosis and in consultation with our haematology colleagues, cervical node ultrasound and biopsy were undertaken in preference to bone marrow biopsy, although this had been discussed with the patient. US appearances showed bilateral multilevel enlarged pathological lymph nodes, the largest at left level 2, measuring 22 mm in maximum diameter. A core biopsy was undertaken for the exclusion of lymphoma.

Repeat EBV serology (day 7 of hospitalization) revealed equivocal EBV VCA IgG (0.77 OD) and positive VCA IgM (9.04 OD). The patient was also found to have developed a new hepatitis (ALT 206 U/L, bilirubin 16 *µ*mol/L, ALP 57 U/L), although he had no clinical features of this. Due to this, a diagnosis of infectious mononucleosis was made and conservative management was instituted alongside dietetic input. By day 11, he had bicytopenia (WCC 3.7 × 10^9^/L, Hb 79 g/L, and Plt 210 × 10^9^/L), and by day 16, he was discharged from the hospital following near total resolution of his symptoms.

Lymph node histopathology reporting in turn highlighted Sternberg-Reed cells and prominent mixed T-cell and B-cell immunoblastic reactions in keeping with likely resolving IM. By day 40 postadmission in outpatient follow-up, he had returned to normal health with his LFTs having normalized and his anaemia now resolved (Hb 132 g/L). Repeat serology taken 110 days after reported symptom onset now showed VCA IgM 3.59OD and VCA IgG (31.82) with negative EBNA IgG confirming recent primary EBV infection.

## 3. Methods

A literature review was conducted in February 2022 using the PubMed and Google Scholar databases. Searches were performed between 2014 and 2022.Keywords included “Epstein-Barr Virus” (EBV) OR “Infectious Mononucleosis” (IM) OR “Glandular Fever” AND “Presentation” OR “complications” OR “Monospot” OR “Heterophile” OR “Serology” OR “Youth”. These terms were used in isolation and in combination to identify search results. From this initial search, 112 research items were identified for review. All abstracts were screened by all authors. Papers were excluded if they were not written in English, duplicitous in nature, not peer reviewed, or had insufficient data regarding the clinicopathology of IM in youth. From these, 45 papers were identified. Following a secondary review of papers, 26 were selected for the final review.

## 4. Discussion

EBV is a ubiquitous herpesvirus of which >90% of adults by age 40 years have been exposed as evidenced by seroprevelance studies with evidence of later stage primary EBV acquisition in children from developed countries [1, 6, 7]. While in many patients there is an excellent prognosis, there are serious complications of note from primary infection of which pneumonia (5–10%), acute liver failure (0.2%), upper airway obstruction (1–3.5%), splenic rupture, and intracapsular bleed (0.1–0.5%) are the most common [2, 5]. More serious complications include, and this is pertinent in this case, haematological complications, which are seen in approximately 25–50% of individuals. These can range from self-limiting aplastic anaemia, thrombocytopenia, agranulocytosis, neutropenia, but also thrombocytopenic thrombotic purpura (TTP), disseminated intravascular coagulation (DIC), and secondary HLH. HLH particularly occurs in EBV in those patients with X-linked lymphoproliferative disease, which has an incidence of 1/1,000,000. HLH is one of the few instances in EBV where use of steroids and other immunosuppressive agents may be indicated. In this case, there was a lack of key diagnostic features from the Yamaguchi criteria including rash, arthritis/arthralgia, or hypofibrinoginaemia [19]. While the serum ferritin was raised at 1024 *µ*g/L equivalent to 1024 ng/mL, it is important to recall that serum ferritin is a nonspecific measure of systemic inflammation and not specific to HLH. In the pivotal HLH-94 study the median ferritin level in secondary HLH was 2950 ng/mL. Indeed, in paediatric studies, ferritin levels of >10,000 ng/mL were 90% sensitive, while in adults, an optimal cut-off of 16,000 ng/mL has been proposed (sensitivity 79.4%, specificity 79.2%, and NPV 98.2%). If higher, further important tests would have included measures of soluble IL-2 receptor level and bone marrow biopsy [19–21]. Other rare complications of IM include neurological features seen in 1–5% of cases, including cerebellitis, optic neuritis, meningoencephalitis (0.5%), and Guillain-Barre syndrome [5, 15].

There is an appreciable relationship between the age of acquisition of EBV and the severity of illness, with children often asymptomatic [5, 6]. The mechanism behind this and the age-related incidence of IM might be due to altered immune responses (i.e., diminished/altered NK cell involvement), which are important in early EBV infection. These immune responses are the predominant method of viral control in children, alongside the involvement of cross-reactive memory T cells, which were created in response to other viral infections and which are highly active but less efficient at clearing the virus [8, 22]. Current epidemiological analysis of EBV in the UK (2002–2013) utilizing Public Health England data shows that there appears to be an increasing trend of hospital admissions for IM, particularly in males and the 15–19 year old age group. It remains unclear why some infected individuals develop IM and others do not. Protective features highlighted from this data against the development of IM included elevated BMI (OR 0.8), non-Caucasian (Black OR-0.21; Asian OR 0.14), and smoking history (OR 0.87), although this was only observed in white individuals. Those with the highest risk of IM included those with lower deprivation, never smoking, and normal/low body weight that has also been documented in American studies [23]. Our patient had therefore several of these at-risk features acquiring IM including being a nonsmoker and Caucasian.

As can be seen, in this case the patient had a severe and prolonged presentation of disease (5 weeks) with recovery expected within several weeks. Indeed, the median duration of odynophagia and lymphadenopathy is 7 and 21 days, respectively [24]. It may be that his severe pancytopenia contributed to his prolonged symptoms of odynophagia, as did his severe iron deficiency, as seen in pathologies such as Plummer-Vinson syndrome [25]. A key confounder was whether the pancytopenia was related to aplastic anaemia secondary to EBV infection. Bone marrow examination, however, was not pursued as per haematology advice and patient preference; however, aplastic anaemia seems likely given the lack of confirmatory blood film examinations as well as the swift improvement in blood counts to intramuscular vitamin B12 and oral folate replacement. The use of corticosteroids in IM is controversial. A 2015 Cochrane review found there to be insufficient evidence to recommend their routine use and alerted to severe complications that occurred with their use including empyema and steroid-induced diabetes. While corticosteroids may have use in the setting of acute airway obstruction secondary to IM, there is no evidence that their routine use reduces the incidence of disease complications, length of stay or readmission rate in IM versus controls and, as such, should not be routinely prescribed [26, 27].

Given the severity of symptoms, it was prudent to perform an US-node and biopsy to exclude underlying lymphoma. Indeed, the presence of Reed-Sternberg cells is common in the setting of IM and in one review of EBV; these were identified in 44% of lymph node biopsies, with all cases revealing immunoblastic proliferation and positive MUM1/IRF4 staining being present in our case [28].

**Figure 1:**
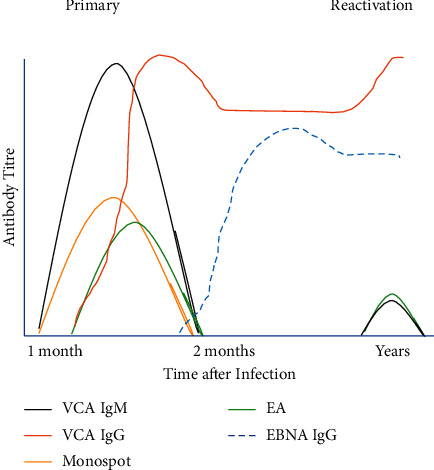
Typical antibody profiles in primary and reactivated EBV infection.

Importantly, this case highlights the need to understand the role of serological tests, which can confirm primary infection when a high index of suspicion exists. The *monospot* test was created in 1932, well before EBV's discovery as the cause of IM and is among the most used. This test is a latex agglutination test which uses equine erythrocytes and tests for heterophile antibodies which are produced during EBV infection by the human immune system [28]. While being specific, its sensitivity is poor in the young (sensitivity 25–50% < 12 years old) [6] with false negatives reported if undertaken early in infection (2–6 weeks). False positives of the monospot can occur in viral hepatitis, rubella, malignancy, HIV, and autoimmune disorders, and in 10% of all patients who are consistently monospot negative [3, 29]. Due to these shortcomings, the monospot test is not routinely used for diagnostic purposes. Current recommendations include the combined use of more specific antibody tests, which are indirect fluorescent tests, which utilise the patient's serum and enable detection of complexes of EBV viral proteins-early antigen (EA), viral capsid antigen (VCA), and Epstein-Barr nuclear antigen (EBNA). The sensitivity of this approach is 95–100% with specificity of 86–90% [6]. Through their use, typical serological profiles can be seen which may delineate primary infection from reactivation (Figure 1). While VCA IgM rises early in the disease process, VCA IgG will peak several weeks after onset. Early antigen (EA) IgG occurs in the acute phase of illness, generally becoming undetectable between 3 and 6 months, during which time the presence of IgG EBNA occurs and persists for life [29, 30].

In our patient, we can see the time-dependent changes in serology with the appearance of the positive monospot coinciding with the presence of IgM VCA and the occurrence of hepatitis, which occurs in 90% of patients. The presence of new hepatitis in the third week of illness, which this was from fever onset returning to normal at six weeks, agrees with a 6-year centre review of EBV hepatitis in 41 immunocompetent patients. This highlights the delayed nature of EBV hepatitis. Moreover, the pathophysiology of EBV hepatitis is not well understood, and it may be given that at this time when the VCA IgM became positive, immune related hepatotoxicity was occurring [31].

The positivity of the monospot normally occurs 4–6 weeks from infection. While not used here, quantitative EBV-DNA measurement can be useful in differentiating between chronic asymptomatic infections where it is undetectable from active EBV disease, particularly within the immunosuppressed population, particularly transplant patients who develop posttransplant lymphoproliferative disorders. It is not recommended for immunocompetent individuals, as it offers no guidance on therapy. Peak viraemia is seen within 2 weeks of postprimary EBV infection with viraemia taking months to years to settle [22].

## 5. Conclusion

In conclusion, this case represents an unusual presentation of IM, compounded by pancytopenia. While the initial presentation was very much in keeping with IM, there was diagnostic uncertainty, particularly concerning the absence of initial hepatitis, the presence of plausible alternate diagnoses, and negative initial monospot and more sensitive serological tests. This case highlights the need to understand the time lag to positivity and deficiencies in serology testing and the need for a high index of suspicion to enable the diagnosis. Physicians should be aware of the atypical features with which IM may present with a propensity for more severe disease in older, white patients who do not smoke.

## Figures and Tables

**Table 1:** Important mimics of glandular fever [2, 11, 15]].

Differentials of glandular fever
Toxoplasmosis
Adenovirus
Influenza
Parainfluenza
Seasonal and novel coronavirus
Herpes simplex virus (HSV)
Acute HIV
